# Parametric Study and Optimization of End-Milling Operation of AISI 1522H Steel Using Definitive Screening Design and Multi-Criteria Decision-Making Approach

**DOI:** 10.3390/ma15124086

**Published:** 2022-06-08

**Authors:** Muhammad Abas, Mohammed Alkahtani, Qazi Salman Khalid, Ghulam Hussain, Mustufa Haider Abidi, Johannes Buhl

**Affiliations:** 1 Department of Industrial Engineering, University of Engineering & Technology, Peshawar P.O. Box 25100, Pakistan; muhammadabas@uetpeshawar.edu.pk (M.A.); qazisalman@uetpeshawar.edu.pk (Q.S.K.); 2 Department of Industrial Engineering, College of Engineering, King Saud University, P.O. Box 800, Riyadh 11421, Saudi Arabia; mabidi@ksu.edu.sa; 3 Mechanical Engineering Department, Faculty of Engineering, University of Bahrain, Isa Town 32038, Bahrain; ghussain@uob.edu.bh; 4 Chair of Hybrid Manufacturing, Brandenburg University of Technology Cottbus-Senftenberg, Konrad-Wachsmann-Allee 17, D-03046 Cottbus, Germany; johannes.buhl@b-tu.de

**Keywords:** end milling, AISI 1522H steel grade, minimum-quantity lubrication (MQL), optimization, combinative distance-based assessment (CODAS), criteria importance through inter-criteria correlation (CRITIC)

## Abstract

End-milling operation of steel grade material is a challenging task as it is hard-to-cut material. Proper selection of cutting tools, cutting conditions, and cutting process parameters is important to improve productivity, surface quality, and tool life. Therefore, the present study investigated the end-milling operation of AISI 1522H steel grade under minimum-quantity lubrication (MQL) conditions using a novel blend of vegetable oils, namely canola and olive oil. Cutting process parameters considered were spindle speed (*s*), feed rate (*f*), depth of cut (*d*), width of cut (*w*), and cutting conditions (*c*), while responses were average surface roughness (Ra), cutting forces (Fc), tool wear (TW), and material removal rate (MRR). Experimental runs were designed based on the definitive screening design (DSD) method. Analysis of variance (ANOVA) results show that feed rate significantly affects all considered responses. Nonlinear prediction models were developed for each response variable, and their validity was also verified. Finally, multi-response optimization was performed using the combinative distance-based assessment (CODAS) method coupled with criteria importance through inter-criteria correlation (CRITIC). The optimized parameters found were: *s* = 1200 rpm, *f* = 320 mm/min, *d* = 0.6 mm, *w* = 8 mm, and *c* = 100 mL/h. Further, it was compared with other existing multi-response optimization methods and induced good results.

## 1. Introduction

In end-milling operation, the key attributes that are highly desirable are lower surface roughness, higher material removal rate, longer tool life, and lower dimensional deviation [1]. These attributes greatly depend on the proper selection of cutting tools, machining conditions, and cutting process parameters, namely cutting speed, feed rate, depth of cut, and width of cut [2]. Dry and nearly dry machining is highly desirable as it is more sustainable than flood machining [3]. In many comparative research studies, MQL performed better than flood machining in surface quality, manufacturing cost, environmental impact, and tool life [3,4,5]. Careful cutting fluid selection is important due to its ecological and human health concerns [4]. Vegetable oil such as olive oil, sunflower oil, and canola oil is proven effective in machining compared to other synthetic lubricants [6]. Vegetable oils are biodegradable and have no harmful effect on interaction with humans [6]. Arsene et al. [5] concluded that, compared to petroleum-based cutting fluids, vegetable oil, i.e., pure corn oil, is more economical and environmentally friendly in MQL-assisted turning of hardened AIDI D2 steel. The study of Kuram et al. [7] revealed that canola cutting fluid is better than sunflower and commercial semi-synthetic cutting fluid in multi-response optimization of surface roughness, tool life, and specific energy during the milling of stainless steel. Yin et al.’s [8] study showed that vegetable oil (i.e., palm, cottonseed, peanut oil, soybean, and castor) performed better in terms of surface roughness and cutting forces than synthetic oil in the milling of AISI 1045 steel.

Optimization of end-milling cutting process parameters is an important step on the production floor. Kuram et al. [7] optimized the cutting process parameters and cutting fluids (vegetable oil (refined sunflower oil or canola and a mixture of emulsifier(s)) in the end milling of AISI 304 steel using a D-optimal design of experiments. They concluded that cutting speed, depth of cut, and feed rate at a higher level reduce the specific energy but increase surface roughness and decrease tool life. Babu et al. [9] optimized the end milling of AISI 304 steel using the technique for order of preference by similarity to ideal solution (TOPSIS). The cutting environmental conditions were dry, MQL (olive oil), and flood lubrication (olive oil). The results reveal that cutting environment conditions have a significant effect on surface roughness and tool wear. Mia et al. [10] optimized the MQL flow rate to reduce the surface roughness and cutting forces in the end milling of HRC 40 hard steel using an integrated approach of grey relational analysis (GRA) and Taguchi methods. The type of lubricant used was ISO grade VG-68 oil. The study showed that high cutting speed, low feed rate, and lubricant at a 150 mL/h flow rate provide lower surface roughness and cutting forces. Parashar and Purohit [11] optimized the material removal rate using Taguchi’s dynamic design of experiments for end-milling operations of steel grade EN 19. Their study showed that the maximum material removal rate could be achieved at high cutting speed, high feed rate, and high depth cut. Kanchana et al. [12] optimized the cutting parameters during the end-milling operation of hardened custom 465 steel using multi-response criteria based on an orthogonal Taguchi matrix with grey relational analysis. The results reveal that the depth of cut was the most contributing factor toward cutting force, material removal rate, and tool–chip interface temperature. In contrast, the feed rate was the most contributing to surface roughness. Airao et al. [13] investigated the surface roughness of SUPER DUPLEX 2507 stainless steel in wet (water) and dry machining conditions. Regression analysis showed that feed rate greatly influences the surface roughness, followed by the cutting speed. Moreover, the surface finish achieved in wet machining is better than in dry machining. Nguyen [14] optimized and developed the prediction models for responses such as average surface roughness, specific cutting energy, and material removal rate in the dry milling of SKD61 material. For optimization, the hybrid approach of the Kriging model and archive-based micro-genetic algorithm (AMGA) was applied. The study showed that feed rate was the most influential cutting parameter affecting all responses. It was difficult to identify an optimal global solution for the conflicting responses, i.e., when the surface roughness decreases, the material removal rate decreases, and specific cutting energy increases. Therefore, considering the weights of the responses (depending on industry requirement) is important in simultaneous optimization. Pimenov et al. [15] analyzed the surface quality, tool wear, material removal rate, and energy consumption for AISI 1045 steel during face milling. The results indicate that the optimized milling performance was possible through grey relational analysis for fast manufacturing. Further, nonlinear regression models were obtained for surface roughness, material removal rate, tool life, and cutting power. Daniyan et al. [16] developed the prediction model based on a combination of central composite design and response surface methodology (RSM) for material removal rate in the end-milling operation of aluminum alloy AA6063-T6. The cutting parameters were also optimized using the composite desirability function. Feed rate, depth of cut, and cutting speed were the key contributing cutting parameters toward material removal rate. Arizmendi et al. [17] proposed an analytical approach for the identification of tool parallel axis offset (TPAO) based on the analysis of transition bands created in the topography of surfaces machined by peripheral milling.

Multi-criteria decision-making (MCDM) methods combined with weight assessment methods are widely used and proved to be efficient in multi-objective optimization of machining operations [18]. The most common MCDM methods are the technique for order of preference by similarity to ideal solution (TOPSIS), multi-objective optimization by ratio analysis (MOORA), vise kriterijumska optimizacija i kompromisno resenje (VIKOR), weighted aggregated sum product assessment (WASPAS), additive ratio assessment (ARAS), complex proportional assessment (COPRAS) and stepwise weight assessment ratio analysis (SWARA), and combinative distance-based assessment (CODAS) [18,19]. Determination of weights is important in multi-response optimization problems [20]. They are classified into subjective and objective methods [20]. Subjective-based methods are based on the judgment of experts. Delphi method, pairwise comparison (such as analytical hierarchy process (AHP)), ranking method, point allocation, and simple multi-attribute rating technique (SMART) are examples of subjective weights [2]. However, no expert’s opinion is required in objective-based methods, and the weights are computed based on available data. The subjective-based methods are standard deviation, entropy, principal component analysis (PCA), and criteria importance through inter-criteria correlation (CRITIC) [2,20]. CRITIC is considered more reliable than other techniques because, unlike other techniques, it incorporates the degree of contrast and conflict in the determination of weights [21]. For instance, Sivalingam et al. [22] compared CODAS and ARAS methods in identifying the optimal parameters for turning of Inconel 718 alloy. They concluded that identical optimal parameters were obtained based on both methods. Abas et al. [23] optimized the turning operation of aluminum alloy 6026-T9 using an integrated approach of MOORA and CRITIC. Further, they concluded that the proposed method performed better than TOPSIS, grey relational analysis, and composite desirability function. Sharsar et al. [24] computed the optimal process parameters of EDM operation based on two approaches, i.e., the integrated approach of entropy with complex proportional assessment (COPRAS) and TOPSIS. The study revealed that the two methods produced similar optimal process parameters. Pandiyan et al. [25] optimized the electrical discharge machining of AA6061-T6/15 wt.% SiC composite using entropy method weights coupled with combinative distance-based assessment (CODAS). Kumar et al. [26] applied an integrated approach of AHP-ARAS for process parameter optimization on EDM machining of AA7050-10%B4C composite. Rao et al. [27] optimized the EDM process parameters for machining AISI D2 steel using the TOPSIS–AHP method. Kalyanakumar et al. [28] optimized the multiple responses based on the VIKOR approach in drilling operations, and Sankar et al. [29] used it for multi-objective optimization in abrasive water jet machining. Sahoo et al. [30] optimized the surface roughness and tool vibration in turning aluminum alloy 6063-T6 by applying the WASPAS method. Singaravel et al. [31] performed multi-objective optimization using multi-objective optimization by ratio analysis (MOORA) and entropy method in turning EN25 steel.

Definitive screening design (DSD), a three-level fractional factorial design, has been developed recently [32]. It performed better than other traditional experimental designs, such as full factorial design and response surface methodology (RSM), in estimating the main effect, interaction, and quadratic effect [33,34]. Compared to traditional methods, it reduces the experimental runs and, therefore, reduces experimentation time and cost. For instance, there are 243 experimental runs for a problem having five factors at three levels using full factorial design and 32 and 46 for RSM based on central composite design and Box–Behnken; however, for DSD, there are a total of 13 experimental runs. Mohammad et al. [35] successfully modeled the effect of various fused deposition modeling (FDM) process parameters on the creep and recovery behavior of 3D printed parts using DSD. In another study [33], they modeled and optimized the dimensional accuracy of FDM parts using DSD and deep learning. Luzanin et al. [36] investigated the effect of build parameters of FDM on the flexural force of FDM manufactured parts. Movrin et al. [37] built the experimental runs based on DSD to optimize vacuum-assisted post-processing of binder jetted specimens. However, its application in end-milling cutting parameter analysis and its optimization is very limited. Therefore, the present study also covered this research gap.

The presented study provides a comprehensive insight into the influence of cutting process parameters on the surface roughness, cutting forces, tool wear, and material removal rate of AISI 1522H steel. This work is particularly interesting for the manufacture of pressure vessels, boilers, heat exchangers, gas turbines, furnaces, and nuclear power plants. This study will help in generating detailed machining data related to steel alloys and can be used as a benchmark to compare other materials. Further, the application of vegetable oil based on a blend of olive and canola oil has not been studied so far in the end milling of steel grade. The blend was also selected because of its good cold flow properties, because it is environment-friendly, biodegradable, and economical, and because it has very low carbon emissions and good lubricating properties. Definitive screening design (DSD) is found effective in the development of nonlinear models and optimization of other manufacturing processes as discussed in the above literature. However, concerning cutting parameters investigation and its optimization, the studies are very limited. DSD is a more economical experimental design than factorial design, Taguchi design, and response surface methodology due to its estimation of a nonlinear model using fewer experimental runs. For multi-objective optimization in end-milling operations of AISI 1522H steel grade, the combinative distance-based assessment (CODAS) method coupled with criteria importance through inter-criteria correlation (CRITIC) has not been utilized and compared with other multi-criteria decision-making (MCDM) methods.

## 2. Experimental Procedure

**Figure 1:**
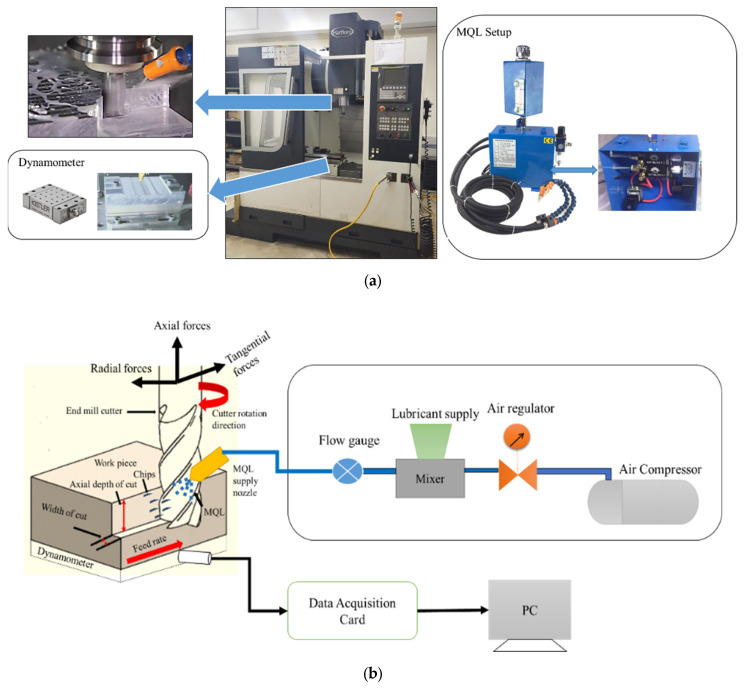
Experimental setup of end-milling machining under minimum-quantity lubrication (MQL). (**a**) Actual experimental setup, (**b**) schematic of the experimental setup.

The end-milling experiments were performed using the CNC machine LG-500 HARTFORD (Hartford machining centers, Shanghai, China). The working material was AISI 1522H steel (Unitedsteel Carbon Plate, Zhengzhou, China) of 200 mm × 200 mm × 10 mm^3^. The chemical properties are shown in Table 1. AISI 1522H steel shows excellent resistance to crevice cracking, chloride pitting, seawater, and heat. It maintains its high strength at elevated temperatures. Because of these properties, it is used extensively in furnaces, gas turbines, pressure vessels, boilers, chemical processing plants, nuclear power plants, and marine engineering. An uncoated four-flute carbide flat end mill cutter (model: SEME71160E, supplied by QINGDAO YG-1 TOOL CO., LTD, Huangdao District, Qingdao, China) having a diameter of 16 mm was used for machining. The minimum-quantity lubrication (MQL) setup used was MQL-2251A-40L1PBM (YONGSHENGHETUO, Guangdong, China). Figure 1 shows the experimental setup and its schematic.

A blend of vegetable oil, namely olive and canola oil (Meezan Group, Karachi, Pakistan), was used as a lubricant because of its biodegradability and good lubricating properties having a relative density of 0.910–0.920 g/cm^3^ at 20 °C, the kinematic viscosity of 79 mm^2^/s at 20 °C, and specific heat of 1.905–1.978 J/g at 20 °C and boiling point 250 °C. They are supplied at 5-bar using a single nozzle inclined at 45°. Experimental runs were designed based on a definitive screening design (DSD). Cutting process parameters and their levels were selected based on literature review, initial experimental runs, and according to a recommendation made by the tool manufacturer. The selected machining parameters, cutting conditions, and their levels are shown in Table 2.

### 2.1. Measurement of Responses

In the present study, four responses were considered to evaluate the cutting performance, i.e., surface roughness, cutting forces, tool wear, and material removal rate. Three consecutive runs were made for each response measurement to obtain its average values to reduce errors.

**Figure 2:**
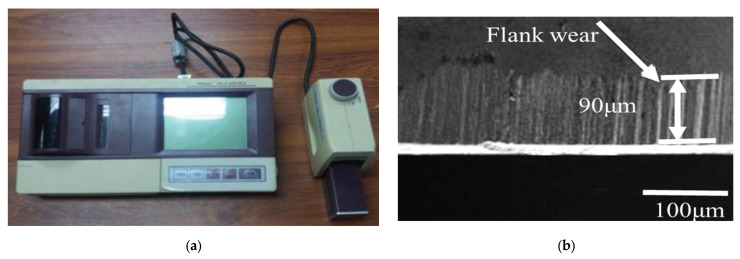
(**a**) Surface roughness testing machine; (**b**) SEM of tool wear.

Surface roughness was measured using the Mitutoyo surface roughness tester (SJ-301, Mitutoyo Corporation, Kanagawa, Japan), as shown in Figure 2a. The average surface roughness profile (Ra) was selected because it is a representative index of machined surface quality and is acceptable in the industry. The Kistler 9257B dynamometer (Kistler, Winterthur, Switzerland) measured cutting forces with the Kistler multichannel charge amplifier type 5070A (Kistler, Winterthur, Switzerland) shown in Figure 1. The present study considered resultant cutting forces (Fc) for further analysis. Tool wear was measured using scanning electron microscopy (SEM) based on ISO 8688-2:1989 standard [38]. According to this standard, the tool failure occurs when average flank wear (V_B_) becomes less or equal to 300 μm or maximum flank wear (V_Bmax_) becomes equal to or less than 600 μm. Figure 2b shows the average flank wear of the tool at experimental run 5, as tabulated in Table 3. The material removal rate (MRR) was computed using the loss weight method using Equation (1) [1].

(1) $$\mathrm{MRR}=\frac{W_{b}-W_{a}}{\rho \times t_{m}}$$

where $W_{b}$ and $W_{a}$ represent the weight of the specimen before and after machining, ρ represents the density of the material and $t_{m}$ the machining time. Experimental runs made based on a definitive screening design and desired measured responses are tabulated in Table 3.

### 2.2. Optimization Methodology

**Figure 3:**
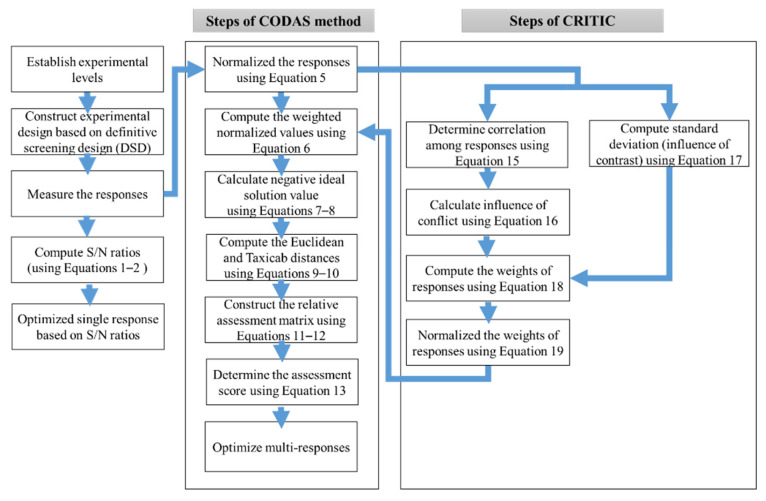
Methodology for single- and multi-response optimization.

Figure 3 shows the steps followed for single- and multi-response optimization using Taguchi signal-to-noise (S/N) ratios and the combinative distance-based assessment (CODAS) method coupled with criteria importance through inter-criteria correlation (CRITIC). S/N ratios control the deviation in the quality characteristics of responses from the desired values [23]. If the response is to be minimized, then the smaller-the-better quality is utilized using Equation (2); however, for maximization, Equation (3) can be applied.

(2) $$\frac{S}{N}\mathrm{ratio}=-10\times {log}_{10}\left(\frac{1}{n}\overset{n}{\underset{i=1}{\sum }}y_{i}^{2}\right)$$

(3) $$\frac{S}{N}\mathrm{ratio}=-10\times {log}_{10}\left(\frac{1}{n}\overset{n}{\underset{i=1}{\sum }}\frac{1}{y_{i}^{2}}\right)$$

where $y_{i}$ represents the response value obtained for experimental run i, and n is the number of repeated experiments.

In practice, simultaneous optimization is highly desirable when conflicting objective functions exist. The conflictive objective functions in the present study are maximization of material removal rate and minimization of average surface roughness, cutting forces, and tool wear. For this aim, the combinative distance-based assessment (CODAS) method coupled with criteria importance through inter-criteria correlation (CRITIC) was applied in the present study. The steps followed for CODAS were adopted from the study of Keshavarz Ghorabaee et al. [39] as follows.

***Step 1*:** Construct decision matrix D having the order n×m of measured responses (m) corresponding to each experimental run (n) having a combination of different levels as tabulated in Table 3 using Equation (4).

(4) $$D_{}={\left[d_{ij}\right]}_{n\times m}=\left[\begin{array}{cccc} d_{11} & d_{12} & \ldots & d_{1n}\\ d_{21} & d_{22} & \ldots & d_{2n}\\ \vdots & \vdots & \ddots & \vdots \\ d_{m1} & d_{m2} & \ldots & d_{mn} \end{array}\right]$$

***Step 2*:** Normalize the decision matrix using Equation (5).

(5) $$\eta_{ij}=\;\;\left\{\begin{array}{l} \frac{d_{ij}}{\underset{i}{\max}d_{ij}},\;if\;j\in M_{b}\\ \frac{\underset{i}{\min}d_{ij}}{\underset{}{d_{ij}}},\;if\;j\in M_{c} \end{array}\right.$$

where $M_{b}\;$ and $M_{c}$ represent the benefit and cost criteria.

***Step 3*:** Compute the weighted normalized decision matrix using Equation (6).

(6) $$\tau_{ij}=\;\lambda_{j}\eta_{ij}$$

where $\lambda_{j}$ is the weight of response such that $\overset{n}{\underset{j=1\;}{\sum }}\lambda_{j}$ = 1.

***Step 4*:** Calculate the negative-ideal solution using Equations (7) and (8).

(7) $$\begin{array}{l} NI\;=\;\left[\varphi_{j}\right]{\;}_{1\times m} \end{array}$$

(8) $$\varphi_{j}=\underset{i}{\min}\tau_{ij}$$

***Step 5*:** Compute the Euclidean and Taxicab distances of experimental runs from the negative-ideal solution using Equations (9) and (10).

(9) $$\varepsilon_{i}=\sqrt{\sum_{j=1}^{m}{\left(\tau_{ij}-\varphi_{j}\right)}^{2}}$$

(10) $$\Gamma_{i}\;=\;\sum_{j=1}^{m}\left|\tau_{ij}-\varphi_{j}\right|$$

***Step 6*:** Construct the relative assessment matrix using Equations (11) and (12).

(11) $$R={\left[\Omega_{ik}\right]}_{n\times n}\;\;where\;\;k\;=\;1,2,\ldots n$$

(12) $$\Omega_{ik}\;=\;\left(\varepsilon_{i}-\varepsilon_{k}\right)+\left(\kappa \left(\varepsilon_{i}-\varepsilon_{k}\right)\times (\Gamma_{i}-\Gamma_{k})\right)$$

where κ is the threshold parameter having a value between 0.01 and 0.05. For the present study, it is set at 0.02 for calculation as suggested in the studies.

***Step 7*:** Determine the assessment score of each experimental run using Equation (13).

(13) $$\;\chi_{i}\;=\sum_{k=1}^{n}\Omega_{ik}$$

***Step 8*:** Rank the experimental run according to the decreasing values of the assessment score. The experimental run (having a combination of different cutting parameter levels) with the highest assessment score represents the best experimental run. However, the optimal cutting parameter levels can be obtained by determining the average values of the assessment score at each level for each factor. For instance, for cutting parameters such as cutting speed $\left(s\right)$ at level 1, the average values can be computed using Equation (14).

(14) $${\left(\rho_{i}\right)}_{1}^{s}\;=\;\frac{{\left(\chi_{i}\right)}_{1}+{\left(\chi_{i}\right)}_{8}+{\left(\chi_{i}\right)}_{11}+{\left(\chi_{i}\right)}_{12}+{\left(\chi_{i}\right)}_{13}}{5}$$

where ${\left(\chi_{i}\right)}_{1}+{\left(\chi_{i}\right)}_{8}+{\left(\chi_{i}\right)}_{11}+{\left(\chi_{i}\right)}_{12}+{\left(\chi_{i}\right)}_{13}$ shows the assessment score for level 1 at experimental runs 1, 8, 11, 12, and 13. Similarly, they were computed for other levels and cutting parameters. The highest value of the average assessment score among three levels for each cutting parameter corresponds to optimal levels.

Weights of responses were determined based on CRITIC. The steps for CRITIC are as follows:

***Step 1*:** Determine the correlation among the normalized responses (obtained in step 2 of the CODAS method) using Equation (15).

(15) $$\alpha_{jk}=\frac{\sum_{i=1}^{m}\left(\mu_{ij}-\bar{\mu_{j}}\right)\left(\mu_{ik}-\bar{\mu_{k}}\right)}{\sqrt{\sum_{i=1}^{m}{\left(\mu_{ij}-\bar{\mu_{j}}\right)}^{2}\sum_{i=1}^{m}{\left(\mu_{ik}-\bar{\mu_{k}}\right)}^{2}}}$$

***Step 2*:** Compute the degree of conflict by applying Equation (16).

(16) $$\upsilon_{j}=\overset{m}{\underset{k=1}{\sum }}\left(1-\alpha_{jk}\right)$$

***Step 4*:** Compute the degree of contrast (standard deviation) using Equation (17).

(17) $$\sigma_{j}=\sqrt{\frac{\sum_{i=1}^{m}{\left(\mu_{ij}-\mu_{j}\right)}^{2}}{m}}$$

***Step 5*:** Combine both degrees of conflict and contrast to obtain weights of responses using Equation (18).

(18) $$\zeta_{j}=\upsilon_{j}\sigma_{j}$$

where $\zeta_{j}$ represents the information emitted (i.e., weights); higher values of $\zeta_{j}$ represent a higher response weight.

***Step 6*:** Finally, normalize the weights using Equation (19).

(19) $$\lambda_{j}=\frac{\zeta_{j}}{\sum_{k=1}^{m}\zeta_{k}}$$

**Figure 4:**
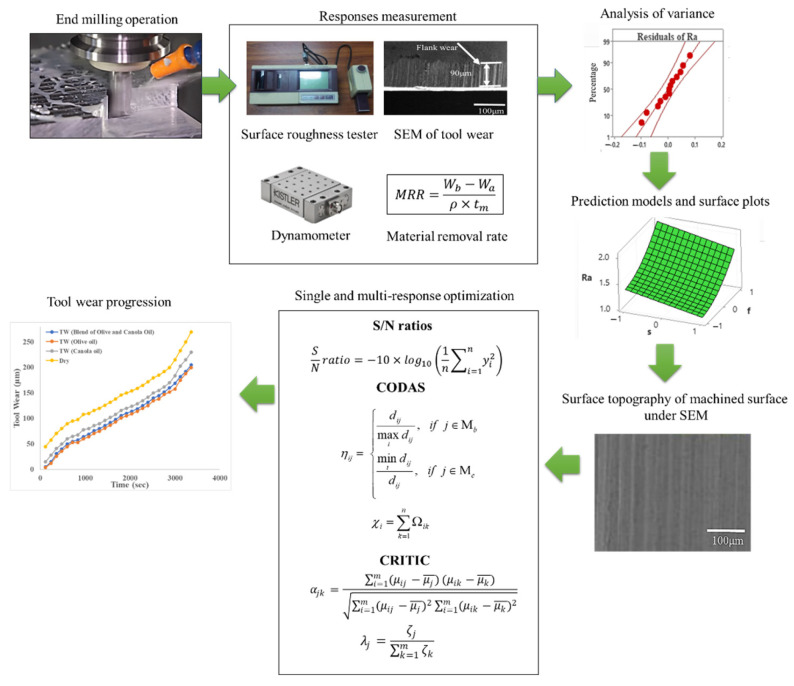
Graphical abstract of present study.

Figure 4 summarizes the present research work.

## 3. Results and Discussions

### 3.1. Development of Models for Responses and Analysis of Variance (ANOVA)

To study the relationship between cutting parameters and responses (i.e., average surface roughness (Ra), cutting forces (Fc), tool wear (TW), and material removal rate (MRR)), second-order regression models using definitive screening design (DSD) were developed based on available data. Coded (−1, 0, 1) cutting process parameters were used as presented in Table 2. As there were 13 experimental runs and each cutting parameter had three levels, there were not enough degrees of freedom to estimate all the terms (i.e., main, interaction, and quadratic) for models. Thus, forward selection based on Bayesian information criteria (BIC) was applied to estimate the model terms. The reduced DSD regression models obtained for Ra, Fc, TW, and MRR are expressed in Equations (20)–(23).

(20) $$\mathrm{Ra}=1.4197-0.1496\times s+0.3322\times f-0.0534\times c+0.1455\times f^{2}-0.1209\times c^{2}-0.0524\times s\times c$$

(21) $$\mathrm{Fc}=976.7-43.5\times s-222.7\times f+194.8\times d+112.9\times w-92.9\times d^{2}-111.5\times w^{2}+152.2\times f\times d+82.7\times d\times w$$

(22) $$\mathrm{TW}=149.33+39.20\times s+32.60\times f+15.40\times d+9.60\times w-14.80\times c+26.47\times s^{2}+16.88\times s\times f$$

(23) $$\mathrm{MRR}=1421.7+232.30\times s+159.10\times f+282.80\times d+106.60\times w+29.50\times c-192.1\times s^{2}-105.8\times d^{2}-97.3\times w^{2}-20.8\times s\times c-110.1\times f\times w$$

Equations (20)–(23) are in good agreement with the literature. For instance, Mia et al. [10] obtained a quadratic equation for surface roughness and cutting force in the end milling of hardened steel (HRC 40). Pimenov et al. [15] obtained nonlinear equations for surface quality, material removal rate, tool life, and cutting power in the end milling of 1045 steel. Airao et al. [13] in their study obtained quadratic models for surface roughness under dry and MQL for Super Duplex 2507 Stainless Steel. Quadratic regression models were obtained in the study of Kuntoğlu et al. [40] for cutting force and material removal rate for the turning of AISI 5140 steel.

The adequacy and significance of these models were analyzed based on the coefficient of determination (R^2^), adjusted R^2^, and predicted R^2^. Regression ANOVA shows the statistical significance (*p*-value) and contribution of most influencing cutting parameters (their main effect, interaction, and quadratic term) to individual responses based on the F-value. A *p*-value less than or equal to the alpha value of 0.05 shows statistical significance. ANOVA was performed for all responses at a 95% confidence interval. The results of reduced regression ANOVA using forward selection based on Bayesian information criteria (BIC) are tabulated in Table 4, Table 5, Table 6 and Table 7. For all responses (i.e., average surface roughness, cutting forces, tool wear, and material removal rate), the overall regression models were found significant, showing that the considered cutting parameters explain the variation in responses as shown in Table 4, Table 5, Table 6 and Table 7.

For average surface roughness, as shown in Table 4, the most influencing cutting parameters were feed rate $\left(f\right)$ followed by spindle speed $\left(s\right)$, quadratic term of feed rate $\left(f^{2}\right)$, and cutting conditions $\left(c^{2}\right)$ with an F-value of 209.640, 85.860, 7.110, and 6.260. These terms were also found statistically significant as *p*-values were less than 0.05. However, the depth of cut $\left(d\right)$ and width of cut $\left(c\right)$ were the least contributing factors and were not estimated by the model. In Table 5, the highest contributing and significant cutting parameters for cutting forces were  f, followed by d, f×d, w, and d×w. Conversely, s and c  were found insignificant. As shown in Table 6, the tool wear’s most contributing and influencing cutting parameters were s, f, d, s×f, c, $s^{2}$, and w. Finally, Table 7 for material removal rate was d, followed by s, f, w, $s^{2}$ and f×w, $d^{2}$, $w^{2}$, c, and s×c. ANOVA results are in line with the literature. For instance, the study of Selvaraj [41] revealed that the feed rate has a significant effect on the cutting force, followed by the spindle speed and the axial depth in the milling operation of 5A grade duplex stainless steel alloy. Mia et al. [10] concluded that MQL significantly affects surface roughness, while spindle speed and feed rate have significant cutting forces in the end milling of HRC 40 hard steel. Parashar and Purohit [11] determined that feed rate followed by the depth of cut were the key parameters affecting the MRR in the end milling of steel grade EN 19. Kumar et al. [42] found that feed rate and spindle speed contribute more to material removal rate and surface roughness in milling AISI 1005 carbon steel. Babu et al. [9] presented that cutting conditions have a significant effect on surface roughness and tool wear.

R^2^ and adjusted R^2^ show a good fit of models to experimental values for all responses, i.e., 98% and 96% for average surface roughness, 98% and 95% for cutting forces, 99% and 97% for tool wear, and 99% and 98% for material removal rate, as tabulated in Table 4, Table 5, Table 6 and Table 7. Predicted R^2^ of 90% for surface roughness, 88% for cutting forces, 91% for tool wear, and 97% for material removal rate exhibit good approximation of models for new experimental runs in defined levels as shown in Table 2.

**Figure 5:**
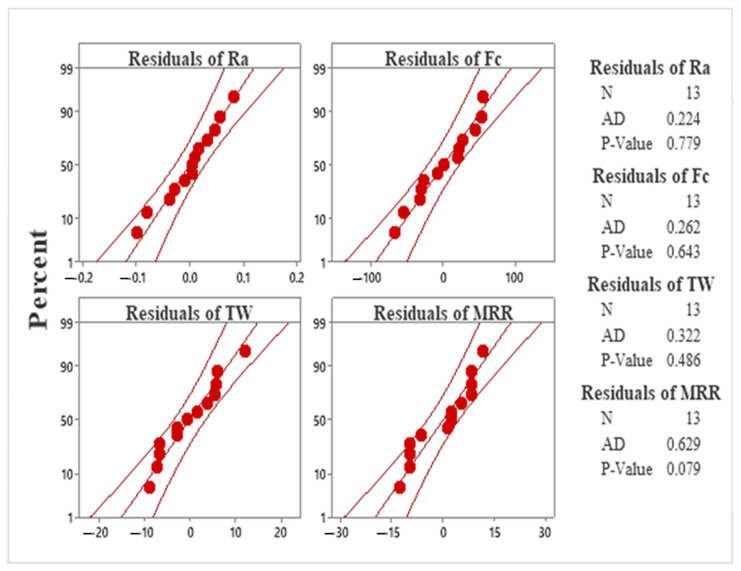
Normal probability plots of residuals for surface roughness, cutting forces, tool wear, and material removal rate.

ANOVA suitability was analyzed using a normal distribution of residuals. As shown in Figure 5, the residuals for all responses, i.e., Ra, Fc, TW, and MRR, fall near the fitted line and were therefore normally distributed [43]. Additionally, the Anderson–Darling test for normality further confirms that the data were normality distributed for all responses as the test’s *p*-values were greater than 0.05. Therefore, it is concluded that the experimental data and developed models are reliable for further investigation and optimization.

### 3.2. Surface Plots of Responses

**Figure 6:**
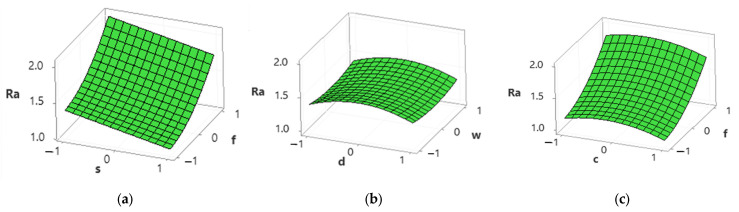
Surface plots for average surface roughness. (**a**) Spindle speed vs. feed rate; (**b**) depth of cut vs. width of cut; (**c**) cutting conditions vs. feed rate.

**Figure 7:**
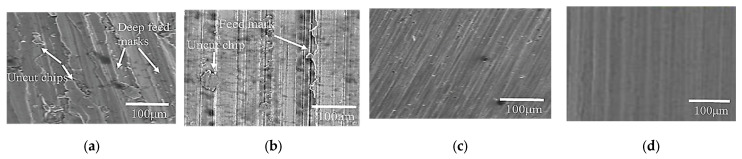
SEM images of machined surface at (**a**) *s* = 900 rpm, *f* = 360 mm/min, *d* = 0.6 mm, *w* =12 mm, *c* = 0 mL/h (dry condition), (**b**) *s* = 900 rpm, *f* = 360 mm/min, *d* = 0.6 mm, *w* =12 mm, *c* = 100 mL/h, (**c**) *s* = 1500 rpm, *f* = 320 mm/min, *d* = 0.2 mm, *w* = 4 mm, *c* = dry condition, (**d**) *s* = 1500 rpm, *f* = 320 mm/min, *d* = 0.2 mm, *w* = 4 mm, *c* = 100 mL/h.

Surface plots were created to understand further the effect of cutting parameters on responses, as shown in Figure 6 and Figure 7. Figure 6 shows that the average surface roughness decreases with an increase in cutting speed and flow rate of lubricant (i.e., cutting conditions); however, it increases drastically with an increase in feed rate. A slight increase was observed with an increase in the depth of cut and width of cut, as shown in Figure 6b. According to Pimenov et al. [15], a higher feed rate and depth of cut leave uncut chips and increase the friction between tool and workpiece interaction and therefore cause poor surface roughness. However, the surface roughness is higher in dry conditions than in MQL conditions because of a reduction in temperature at the tool–chip interface, and chips are removed easily due to the MQL system [44]. It can be justified further by the SEM images as shown in Figure 7a,b. Figure 7a shows deep feed marks and uncut chips at low cutting speed, high feed rate, high depth of cut, and high width of cut under dry conditions, while in the MQL condition, the surface finish is relatively smooth with feed marks and slight uncut chips as shown in Figure 7b. Higher cutting speed reduces steel’s hardness, therefore reducing the surface roughness [40]. Figure 7c,d show the SEM images of the surface roughness at high cutting speed and low feed rate, low depth of cut, and low width of cut under dry and high MQL level.

**Figure 8:**
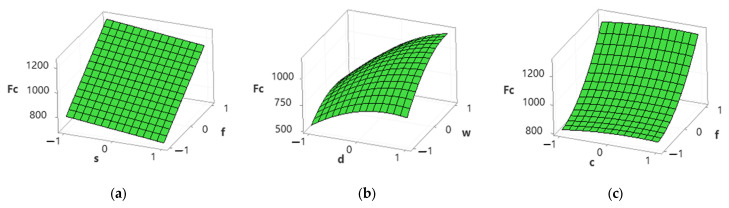
Surface plots for cutting forces. (**a**) Spindle speed vs. feed rate; (**b**) depth of cut vs. width of cut; (**c**) cutting conditions vs. feed rate.

Figure 8 shows that the decrease in cutting forces was observed with an increase in cutting speed, but it increased with an increase in feed rate, depth of cut, and width of cut. No explicit trends were observed for cutting conditions, as shown in Figure 8c. Mia et al. [10] stated that the application of MQL has little effect on cutting forces. Higher cutting speed reduces the rubbing of tools and chips and minimizes the shear section, resulting in lower cutting forces [45]. According to Kannan et al. [46], the other possible reason is the reduction in built-up edges at a high cutting speed that reduces cutting forces. An increase in feed rate and depth of cut increases the chip cross-section and shear area and therefore increases the cutting force [47,48].

**Figure 9:**
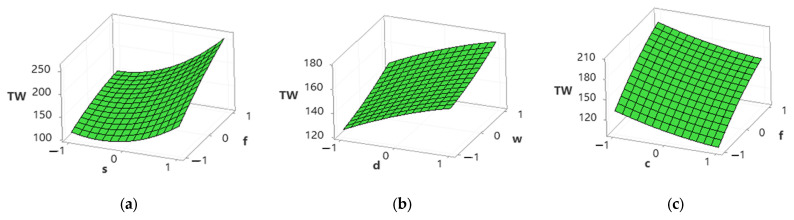
Surface plots for tool wear. (**a**) Spindle speed vs. feed rate; (**b**) depth of cut vs. width of cut; (**c**) cutting conditions vs. feed rate.

Figure 9 shows that tool wear increases with an increase in cutting speed, feed rate, depth of cut, and width of cut; however, it decreases with an increase in cutting conditions. An increase in cutting speed, feed rate, depth of cut, and width of cut increases the temperature at the tool–work interface and affects the hardness of the cutting tool and therefore accelerates the tool wear rigorously [49,50]. However, the tool wear rate is higher in dry conditions than in MQL conditions due to a decrease in temperature at the cutting zone, and also, the chips are flushed at high speed due to the MQL system [2,49,50].

**Figure 10:**
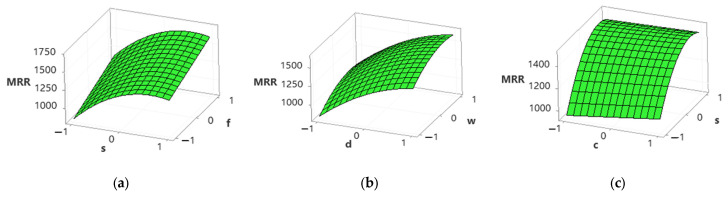
Surface plots for material removal rate. (**a**) Spindle speed vs. feed rate; (**b**) depth of cut vs. width of cut; (**c**) cutting conditions vs. feed rate.

Figure 10 shows an increase in material removal rate (MRR) with an increase in cutting speed, feed rate, depth of cut, width of cut, and cutting conditions. An increase in cutting speed, feed rate, and depth of cut causes plastic deformation and thermal softening of material and therefore increases MRR [51]. The chip thickness under MQL conditions is relatively smaller than in dry conditions, attributed to a reduction in temperature and adhesion between the cutting tool and chip [52]. Moreover, MQL causes chip breaking easily and improves the MRR [1,52].

### 3.3. Single-Response Optimization Based on S/N Ratios

The responses were optimized individually using signal-to-noise (S/N) ratios. For average surface roughness (Ra), cutting forces (Fc), and tool wear (TW), the smaller-the-better-quality characteristic based on S/N ratios was applied using Equation (2). For material removal rate (MRR), the larger-the-better-quality characteristic based on S/N ratios was employed using Equation (3). The results are tabulated in Table 8. Larger values of S/N ratios indicate an excellent performance of responses. For Ra, the larger value of S/N ratios observed was 0.80 at experimental run 12, having cutting speed $\left(s\right)$, depth of cut $\left(d\right)$ and cutting conditions $\left(c\right)$ at a high level, feed rate $\left(f\right)$ at a low level, and width of cut $\left(w\right)$ at medium level. For Fc, a larger value of S/N ratios observed was −53.33 at experimental run 11 having s and w at high levels, f  and d at a low level, and c at medium level. For TW, a larger value of S/N ratios computed was −39.08 at experimental run 5 having  s, f, and w  at low levels, d  at medium level, and c at a high level. Finally, for MRR, a larger value of S/N ratios was −64.35 at experimental run 3, with d, w, and c  at high levels and s at medium levels.

### 3.4. Multi-Response Optimization

Multi-response optimization was performed based on the combinative distance-based assessment (CODAS) method coupled with criteria importance through inter-criteria correlation (CRITIC), as discussed in Section 2.2. Table 9 shows the normalized, weighted normalized, negative-ideal solution, and distance measurement using Equations (5)–(10). Table 10 depicts a relative assessment matrix with the assessment score using Equations (11)–(13). The highest assessment score value obtained is 1.008 at experimental run 3, and therefore it ranks first. However, to find an experimental run with optimal levels, we computed the average values of the assessment score at each level for each factor as obtained in Table 11. For instance, for cutting parameters such as cutting speed $\left(s\right)$ at level 1, the average value of the assessment score can be computed using Equation (24).

(24) $${\left(D_{i}\right)}_{s,1}=\frac{{\left(D_{i}\right)}_{1}+{\left(D_{i}\right)}_{8}+{\left(D_{i}\right)}_{11}+{\left(D_{i}\right)}_{12}+{\left(D_{i}\right)}_{13}}{5}$$

where ${\left(D_{i}\right)}_{1}+{\left(D_{i}\right)}_{8}+{\left(D_{i}\right)}_{11}+{\left(D_{i}\right)}_{12}+{\left(D_{i}\right)}_{13}$ shows the assessment score value for level 1 at experimental runs 1, 8, 11, 12, and 13. Similarly, average values were computed for other levels and for each cutting process parameter as computed in Table 11. The highest value of the average assessment score among three levels for each cutting parameter corresponds to optimized levels, i.e., 0.383 at level 0 for cutting speed $\;\left(s\right)$, 0.063 for feed rate $\;\left(f\right)$ at level −1, 0.322 for the depth of cut $\left(d\right)$ at level 1, 0.050 for the width of cut (w) at level 1, and 0.134 for cutting conditions $\left(c\right)$ at level 1. At optimized levels, the values of desired cutting parameters are s—1200 rpm, f—320 mm/min, d—0.6 mm, w—12 mm, and c—100 mL/h.

Weights were determined for responses based on the CRITIC method. Table 12 represents the correlation matrix computed from the normalized matrix in Table 9 by applying Equation (15). Degree of contrast ($v_{j}$), conflict $\left(\sigma_{j}\right)$, and weights of individual responses were computed by applying Equations (16)–(19). The highest weight was attributed to MRR (0.41) and therefore ranked first, followed by TW (0.23), Fc (0.22), and Ra (0.14), as shown in Table 12.

### 3.5. Validation Test and Comparative Analysis

Validation tests were performed to verify the adequacy of the developed prediction models in Equations (20)–(23). Five experimental runs for each response variable were made based on levels not included in DSD experimental design. Table 13 shows the summarized results of validation tests. The results show that the predictions made by the developed models are in good agreement with actual values and the percentage error observed for all responses is less than 5%. The optimized parameters obtained based on the proposed method were compared with other methods such as composite desirability, vise kriterijumska optimizacija i kompromisno resenje (VIKOR), technique for order of preference by similarity to ideal solution (TOPSIS), multi-objective optimization by ratio analysis (MOORA), and grey relational analysis (GRA). The results show that for multi-response optimization, CODAS–CRITC performed better than the aforementioned techniques, as shown in Table 14.

**Figure 11:**
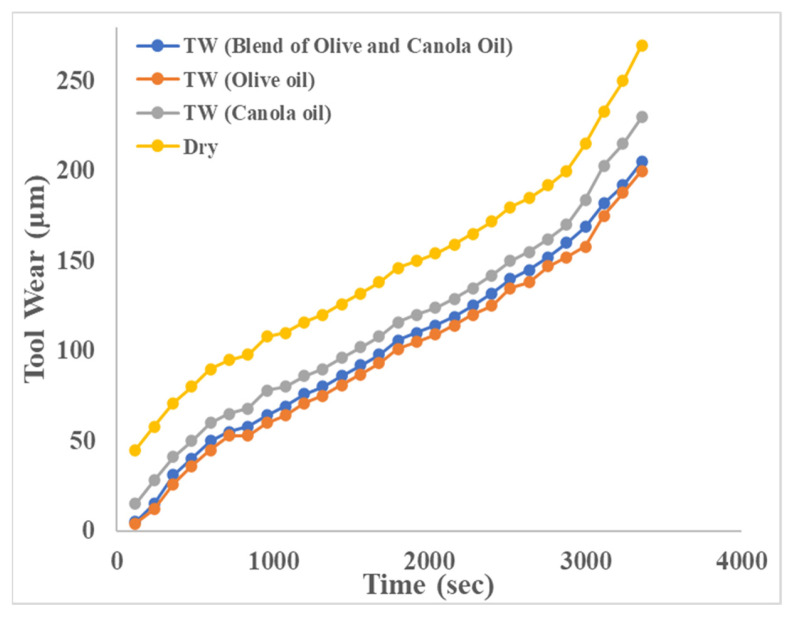
Tool wear progression curve under different machining conditions.

The performance of the blend of canola and olive oil for different responses was also compared with other vegetable oils at optimized parameters, i.e., *s*—1200 rpm, *f*—320 mm/min, *d*—0.6 mm, *w*—8 mm, and *c*—100 mL/h, as shown in Table 15. The results show that a blend of olive and canola oil performed better than canola and sunflower while being approximately equivalent to olive oil in terms of average surface roughness, cutting forces, tool wear, and material removal rate. Further, as shown in Figure 11, the tool wear progression curve shows that the development of tool wear in dry machining conditions is relatively higher than in MQL machining conditions. It also indicates that tool wear progression under a blend of olive and canola oil and virgin olive oil is approximately equal.

The good performance of virgin olive oil may be attributed to its deep and stretchy particles that provide greater capability to provide good lubrication and to bear stresses. The molecules of olive oil are polar in nature which allows them to align themselves by opposite charges on poles making the bond strong and smooth to each other on the surface of the metal. Thus strong bonding allows it to perform better in terms of lubricity and durability [53]. The good performance of olive and canola oil over sunflower may be attributed to their long fatty acid chains. Long fatty acid chains can sustain high cutting temperatures and therefore reduce tool wear and improve surface protection [54]. The higher viscosity of a blend of olive and canola oil provides efficient lubrication by resisting the flow of oil at the tool–chip interface. It also reduces the friction between the cutter and workpiece and allows the removal of heat easily at the tool–chip interface [55,56]. Therefore, the blend of olive and canola oil can be used as an alternative to virgin olive oil as it is more economical.

## 4. Conclusions

End-milling operation was performed for AISI 1522H steel alloy under minimum-quantity lubrication (MQL) conditions using a novel blend of vegetable oils, namely canola and olive oil. Based on experimental results and statistical analysis, the following conclusions were drawn:

- Analysis of variance showed that feed rate has a dominant effect on all considered responses, namely surface roughness (Ra), cutting forces (Fc), tool wear (TW), and material removal rate (MRR).
- Definitive screening design (DSD) was found efficient in the reduction of experimental runs (total of 13 experimental runs) and development of prediction models (i.e., quadratic regression models) for considered responses. Through confirmation tests, the validity of individual models was successfully justified.
- Scanning electron microscopy (SEM) of the machined surface showed that machining under minimum-quantity lubrication (MQL) provides a relatively smooth surface compared to dry machining.
- The cutting process parameters were simultaneously optimized based on CODAS–CRITIC and induced better results than other multi-criteria decision-making methods such as composite desirability, TOPSIS, VIKOR, GRA, and MOORA. The optimal parameters obtained were s—1200 rpm, f—320 mm/min, d—0.6 mm, w—8 mm and c—100 mL/h having Ra = 1.101 µm, Fc = 781 N, TW = 151 µm, and MRR = 1540 mm^3^/s.
- The tool wear progression curve showed that tool wear development in dry machining conditions is relatively higher than in MQL machining conditions. It also showed that tool wear progression under a virgin olive oil and canola oil blend is approximately equal. Therefore, the blend of olive and canola oil can be used as an alternative to virgin olive oil as it is more economical.
- The present study will guide improving productivity, cutting tool performance, and good quality machining of AISI 1522H steel.
- This work is particularly interesting for the manufacture of pressure vessels, boilers, heat exchangers, gas turbines, furnaces, and nuclear power plants. This study will help in generating detailed machining data related to steel alloys and can be used as a benchmark to compare other material.
- Future studies can incorporate the effect of other response variables such as dimensional accuracy and energy consumption. Machine learning and neural network can be employed to develop more accurate prediction models.

## Figures and Tables

**Table 1:** Chemical properties of AISI 1522H steel.

Elements	C	Si	Cr	Mn	Ni	P	S	AL	Mo
Max%	0.17–0.23	≤0.40	≤0.40	1–1.5	≤0.40	0.035	0.035	0.02	0.10

**Table 2:** Cutting parameters/conditions and their levels.

Cutting Parameters/Conditions	Symbols	Units	Levels		
			−1	0	1
Spindle speed	s	rpm	900	1200	1500
Feed rate	f	mm/min	320	340	360
Depth of cut	d	mm	0.2	0.4	0.6
Width of cut	w	mm	4	8	12
Cutting conditions	c	mL/h	0	50	100

**Table 3:** Experimental runs based on definitive screening design and measured responses.

Experimental Runs	Coded Cutting Parameters Levels					Responses			
	s	f	d	w	c	Ra (μm)	Fc (N)	TW (μm)	MRR (mm^3^/s)
1	1	1	0	1	−1	1.814	1184	295	1520
2	0	0	0	0	0	1.452	977	151	1423
3	0	1	1	1	1	1.625	1505	185	1675
4	0	−1	−1	−1	−1	1.183	498	112	541
5	−1	−1	0	−1	1	1.252	546	90	565
6	−1	0	−1	1	1	1.495	695	128	682
7	−1	1	−1	0	−1	1.936	748	145	710
8	1	1	−1	−1	1	1.525	594	222	1152
9	−1	1	1	−1	0	2.051	1242	162	1243
10	−1	−1	1	1	−1	1.235	823	158	1085
11	1	−1	−1	1	0	1.047	464	151	1023
12	1	−1	1	0	1	0.912	715	172	1495
13	1	0	1	−1	−1	1.175	662	235	1418

**Table 4:** Regression analysis of variance for average surface roughness.

Source	Degrees of Freedom	F-Value	*p*-Value
Regression Model	6	44.930	0.000
Linear	3	85.860	0.000
s	1	42.510	0.001
f	1	209.640	0.000
c	1	5.420	0.059
Square	2	5.810	0.039
$f^{2}$	1	7.110	0.037
$c^{2}$	1	6.260	0.046
Interactions	1	3.250	0.122
s×c	1	3.250	0.122
Error	6		
Total	12		
R^2^ = 98%, Adjusted R^2^ = 96%, Predicted R^2^ = 90%			

**Table 5:** Regression analysis of variance for cutting forces.

Source	Degrees of Freedom	F-Value	*p*-Value
Model	8	31.02	0.002
Linear	4	52.9	0.001
s	1	3.92	0.119
f	1	102.71	0.001
d	1	78.58	0.001
w	1	26.4	0.007
Square	2	5.01	0.081
$d^{2}$	1	4.03	0.115
$w^{2}$	1	4.47	0.102
Interactions	2	15.4	0.013
f×d	1	27.5	0.006
d×w	1	10.41	0.032
Error	4		
Total	12		
R^2^ = 98%, Adjusted R^2^ = 95%, Predicted R^2^ = 88%			

**Table 6:** Regression analysis of variance for tool wear.

Source	Degrees of Freedom	F-Value	*p*-Value
Model	7	51.3	0.001
Linear	5	63.91	0.001
s	1	155.99	0.001
f	1	107.89	0.001
d	1	24.08	0.004
w	1	9.36	0.028
c	1	22.24	0.005
Square	1	16.41	0.010
$s^{2}$	1	16.41	0.010
Interactions	1	23.13	0.005
s×f	1	23.13	0.005
Error	5		
Total	12		
R^2^ = 99%, Adjusted R^2^ = 97%, Predicted R^2^ = 91%			

**Table 7:** Regression analysis of variance for material removal rate.

Source	DF	F-Value	*p*-Value
Model	10	418.98	0.002
Linear	5	785.09	0.001
s	1	1243.43	0.001
f	1	583.26	0.002
d	1	1816.85	0.001
w	1	261.84	0.004
c	1	20.05	0.046
Square	3	81.1	0.012
$s^{2}$	1	139.42	0.007
$d^{2}$	1	35.83	0.027
$w^{2}$	1	30.3	0.031
2-Way Interactions	2	59.07	0.017
s×c	1	3.16	0.217
f×w	1	107.32	0.009
Error	2		
Total	12		
R^2^ = 99%, Adjusted R^2^ = 98%, Predicted R^2^ = 97%			

**Table 8:** S/N ratios of individual responses.

Experimental Runs	Ra	Fc	TW	MRR
1	−5.17	−61.47	−49.40	63.64
2	−3.24	−59.80	−43.58	63.06
3	−4.22	−63.55	−45.34	64.48 *
4	−1.46	−53.94	−40.98	54.66
5	−1.95	−54.74	−39.08 *	55.04
6	−3.49	−56.84	−42.14	56.68
7	−5.74	−57.48	−43.23	57.03
8	−3.67	−55.48	−46.93	61.23
9	−6.24	−61.88	−44.19	61.89
10	−1.83	−58.31	−43.97	60.71
11	−0.40	−53.33 *	−43.58	60.20
12	0.80 *	−57.09	−44.71	63.49
13	−1.40	−56.42	−47.42	63.03

* Higher S/N ratio values.

**Table 9:** Normalized and distance values of responses.

Experimental Run	Normalized Matrix				Weighted Normalized Matrix				Distances	
	Ra	Fc	TW	MRR	Ra	Fc	TW	MRR	Euclidean	Taxicab
1	0.503	0.392	0.305	0.907	0.088	0.083	0.065	0.362	0.234	0.261
2	0.628	0.475	0.596	0.850	0.110	0.100	0.128	0.339	0.224	0.340
3	0.561	0.308	0.486	1.000	0.099	0.065	0.104	0.399	0.274	0.329
4	0.771	0.932	0.804	0.323	0.136	0.197	0.172	0.129	0.179	0.296
5	0.728	0.850	1.000	0.337	0.128	0.180	0.214	0.135	0.194	0.319
6	0.610	0.668	0.703	0.407	0.107	0.141	0.151	0.162	0.122	0.224
7	0.471	0.620	0.621	0.424	0.083	0.131	0.133	0.169	0.103	0.178
8	0.598	0.781	0.405	0.688	0.105	0.165	0.087	0.274	0.180	0.294
9	0.445	0.374	0.556	0.742	0.078	0.079	0.119	0.296	0.176	0.235
10	0.738	0.564	0.570	0.648	0.130	0.119	0.122	0.258	0.160	0.292
11	0.871	1.000	0.596	0.611	0.153	0.211	0.128	0.244	0.210	0.398
12	1.000	0.649	0.523	0.893	0.176	0.137	0.112	0.356	0.262	0.443
13	0.776	0.701	0.383	0.847	0.136	0.148	0.082	0.338	0.233	0.367
	**Negative-ideal solution (NI)**				**0.064**	**0.068**	**0.069**	**0.133**		

**Table 10:** Relative assessment matrix with assessment score and rank.

Experimental Runs	1	2	3	4	5	6	7	8	9	10	11	12	13	Assessment Score	Rank
1	0.000	0.010	−0.040	0.055	0.040	0.112	0.132	0.054	0.058	0.074	0.024	−0.028	0.001	0.492	3
2	−0.010	0.000	−0.049	0.045	0.030	0.102	0.122	0.044	0.048	0.064	0.014	−0.037	−0.009	0.365	5
3	0.040	0.049	0.000	0.095	0.079	0.151	0.171	0.094	0.098	0.114	0.064	0.012	0.041	1.008	1
4	−0.055	−0.045	−0.095	0.000	−0.015	0.057	0.076	−0.001	0.003	0.019	−0.031	−0.083	−0.054	−0.223	9
5	−0.040	−0.030	−0.079	0.015	0.000	0.072	0.092	0.014	0.018	0.034	−0.016	−0.067	−0.038	−0.024	7
6	−0.111	−0.102	−0.151	−0.056	−0.072	0.000	0.020	−0.057	−0.054	−0.037	−0.087	−0.139	−0.110	−0.956	12
7	−0.131	−0.121	−0.170	−0.076	−0.091	−0.020	0.000	−0.077	−0.073	−0.057	−0.107	−0.158	−0.130	−1.211	13
8	−0.054	−0.044	−0.094	0.001	−0.014	0.057	0.077	0.000	0.004	0.020	−0.030	−0.082	−0.053	−0.212	8
9	−0.058	−0.048	−0.097	−0.003	−0.018	0.054	0.073	−0.004	0.000	0.016	−0.034	−0.085	−0.056	−0.260	10
10	−0.074	−0.064	−0.114	−0.019	−0.034	0.038	0.057	−0.020	−0.016	0.000	−0.050	−0.102	−0.073	−0.471	11
11	−0.024	−0.014	−0.064	0.031	0.016	0.088	0.108	0.030	0.034	0.050	0.000	−0.052	−0.023	0.178	6
12	0.028	0.038	−0.012	0.083	0.068	0.140	0.160	0.082	0.086	0.102	0.052	0.000	0.029	0.855	2
13	−0.001	0.009	−0.041	0.054	0.039	0.111	0.131	0.053	0.057	0.073	0.023	−0.029	0.000	0.477	4

**Table 11:** Optimal levels based on average assessment score.

Cutting Parameters	Levels			Optimal Levels
	−1	0	1	
Cutting speed	−0.585	0.383 *	0.358	0
Feed rate	0.063 *	−0.038	−0.037	−1
Depth of cut	−0.485	0.278	0.322 *	1
Width of cut	−0.049	0.003	0.050 *	1
Cutting conditions	−0.187	0.095	0.134 *	1

* Optimal levels for each cutting parameters.

**Table 12:** Correlation matrix and weight determination of responses based on CRITIC.

Correlation Matrix				
	Ra	Fc	TW	MRR
Ra	1.00	0.60	0.19	−0.03
Fc	0.60	1.00	0.48	−0.66
TW	0.19	0.48	1.00	−0.80
MRR	−0.03	−0.66	−0.80	1.00
CRITIC parameters				
$\nu_{j}$	2.23	2.57	3.12	4.48
$\sigma_{j}$	0.16	0.21	0.18	0.22
$\zeta_{j}$	0.35	0.53	0.55	1.00
$w_{j}$	0.14	0.22	0.23	0.41
Rank	4	3	2	1

**Table 13:** Validation tests of prediction model.

Experimental Runs for Validation	Coded Cutting Parameters	Responses											
	*s*, *f*, *d*, *w*, *c*	Ra			Fc			TWR			MRR		
		A	P	% E	A	P	%E	A	P	%E	A	P	%E
1	0, −1, −1, −1,1	1.11	1.06	4.72	513	500	2.60	83	80	3.75	588	597	1.51
2	1, −1, −1, −1, 1	0.91	0.87	4.60	485	475	2.11	131	127	3.15	632	644	1.86
3	0, 0, 1, −1, −1	1.4	1.35	3.70	772	790	2.28	174	170	2.35	1342	1360	1.32
4	1, 0, −1, 0, 1	1.09	1.05	3.81	741	723	2.49	181	185	2.16	1072	1089	1.56
5	−1, −1, −1, 0, −1	1.32	1.26	4.76	643	657	2.13	124	120	3.33	401	406	1.23
Actual (A), Predicted (P), and Error (E)													

**Table 14:** Comparison of CODAS–CRITIC with other methods.

Methods	Optimized Parameters (*s*, *f*, *d*, *w*, *c*)	Ra (μm)	Fc (N)	TW (μm)	MRR (mm^3^/s)
Composite Desirability	1, −1, 1, 0, 0	1.015	720	174	1482
VIKOR	1, −1, 1, 0, 0	1.015	720	174	1482
TOPSIS	1, −1, −1, 0, 1	1.032	458	154	1014
MOORA	1, −1, −1, 0, 1	1.032	458	154	1014
GRA	0, 1, 1, 1, 1	1.625	1505	185	1675
CODAS–CRITIC	0, −1, 1, 1, 1	1.101	781	151	1540

**Table 15:** Comparison of vegetable oils at optimized levels for responses.

Vegetable Oil	Ra (μm)	Fc (N)	TW (µm)	MRR (mm^3^/s)
Blend of olive and canola oil	1.101	781	151	1540
Olive oil	1.022	778	147	1551
Canola oil	1.125	787	156	1535
Sunflower oil	1.131	791	163	1525

## Data Availability

Not applicable.
